# Microfluidic Platforms for High-Throughput Pancreatic Ductal Adenocarcinoma Organoid Culture and Drug Screening

**DOI:** 10.3389/fcell.2021.761807

**Published:** 2021-12-23

**Authors:** Marlene Geyer, Karla Queiroz

**Affiliations:** MIMETAS BV, Oegstgeest, Netherlands

**Keywords:** PDAC, tumor microenvionment, drug screening, organoids, organ-on-a chip

## Abstract

Pancreatic Ductal Adenocarcinoma (PDAC), the most common pancreatic cancer type, is believed to become the second leading cause of cancer-related deaths by 2030 with mortality rates of up to 93%. It is often detected at a late stage due to lacking symptoms, and therefore surgical removal of the tumor is the only treatment option for patients. Only 20% of the tumors are resectable, mainly due to early metastasis. Therefore, for 80% of cases chemotherapeutic treatment is the leading therapy for patients. PDAC is characterized by high-density stroma which induces hypoxic conditions and high interstitial pressure. These factors impact carcinogenesis and progression of PDAC and support the formation of an immunosuppressive microenvironment that renders this tumor type refractory to immunotherapies. Most *in vitro* PDAC models have limited translational relevance, as these fail to recapitulate relevant aspects of PDAC complexity. Altogether, there is an urgent need for novel and innovative PDAC modeling platforms. Here, we discuss the relevance of microfluidic and organoid technologies as platforms for modeling bio- and physicochemical features of PDAC and as translational models that enable high-throughput phenotypic drug screenings, while also allowing for the development of novel personalized models used to identify treatment responsive patient subsets.

## Pancreatic Ductal Adenocarcinoma

The pancreas consists of three main cell types: acinar cells, which secrete digestive enzymes, duct cells secreting bicarbonate and hormone-secreting endocrine islet cells (Kleeff et al., 2016). The most common pancreatic cancer type involves the exocrine part and is known as pancreatic ductal adenocarcinoma (PDAC). This disease is hard to predict, detect, diagnose, and treat. In 90% of these tumors KRAS codon 12 is mutated, whereas TP53 (“the gatekeeper”), CDKN2A and SMAD4 are mutated with an incidence rate of 50–80% (Kleeff et al., 2016). In addition, epigenetic and copy number variations of genes, as well as somatic and germline mutations including the repair pathways BRCA1/2, ATM and PALB2 are characteristics of PDAC. The TGF- β, WNT, NOTCH, and DNA damage repair pathways are potential drug targets as these are also activated in PDAC. Aerobic glycolysis and pentose phosphate pathways are upregulated in pancreatic cancer stem cells, which represent a minority in the tumor microenvironment making therapeutic targeting of these metabolic pathways difficult, as apart from their self-renewing characteristics these are also of heterogenous nature (Huang et al., 2015).

PDAC is furthermore characterized by resistance to conventional treatments and rapid metastasis to liver, lung, and peritoneal cavity. Both liver and pancreas arise from endoderm and control metabolism by secreting enzymes. Their common developmental origin and function is thought to be one of the reasons pancreatic tumors first metastasize to the liver (Hindley et al., 2016). This propensity to metastasize originates from paracrine and autocrine signals of guiding cells towards other tissue. During metastasis, cancer cells from the primary tumor invade foreign microenvironment facilitated by *KRAS, TP53, p16, CDKN2A* and *SMAD4*. These intravasate into the bloodstream, disseminate, extravasate through the endothelia, enter and colonize a distant organ (Thomas et al., 2020). Epithelial-to-mesenchymal-transition (EMT) plays a role in metastasis, however, only a small number of cells in pancreatic cancer have been shown to undergo EMT and thus the contribution of EMT is not fully understood in PDAC metastasis (Tan et al., 2014).

## A Complex Tumor Microenvironment

**FIGURE 1:**
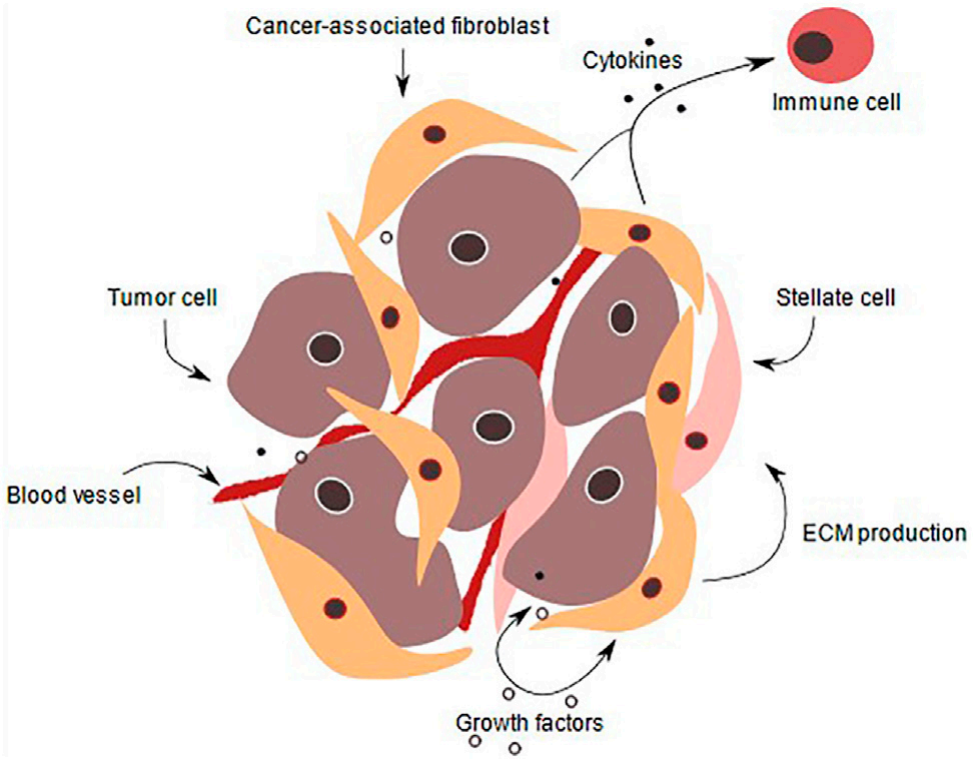
PDAC tumor microenvironment. In a normal pancreas, the basal lamina is highly organized, and apical-basal polarity is present. Tissue is vascularized and ECM supports pancreatic cells. Also, pancreatic stellate cells produce ECM proteins and remain quiescent. During pancreatic intraepithelial lesion, the ductal cells start to transform, their morphology as well as gene expression change. Immune cells are recruited and fibroblasts as well as pancreatic stellate cells become activated, thereby secreting a variety of signaling factors, that are received by the transformed cells. ECM production is enhanced and PDAC is initiated, transformed cells start to form niches and colonize. ECM deposition increases and the stroma amount increases up to 90% of the whole tumor volume. The cancer cells proliferate due to various growth factors secreted by immune cells such as CD8^+^ T-cells, tumor-associated macrophages and pancreatic stellate cells. In addition, they invade other tissue and intravasate leading to metastasis to other organs (Kota et al., 2017).

PDAC cells are supported by a complex microenvironment (Figure 1) that is composed of approximately 90% stroma. This dense stroma creates a hypoxic environment, and mainly consists of collagen, fibronectin, fibrillar collagen and hyaluronic acid (HA). Increased HA content promotes cancer-cell migration and increases the interstitial pressure, which limits drug availability. Enhanced HA production is a prognostic factor in PDAC but also laminin expression correlated with poor patient prognosis, as it increases drug resistance due to promoting high cell adhesion (Miyamoto et al., 2004). Several strategies for targeting HA, such as synthesis inhibition, signal blockage and HA depletion in the stroma have shown beneficial effects in PDAC treatment (Jacobetz et al., 2012; Jiang et al., 2015; Nagy et al., 2015). In addition, the tumor microenvironment (TME) comprises mesenchymal derived cells such as fibroblasts, pericytes, endothelium and immune cells such as T-cells, B-cells, macrophages, dendritic cells (DCs), eosinophils, myeloid-derived suppressor cells (MDSCs) and natural killer cells (NK-cells) (Palucka et al., 2016).

In particular, tumor associated macrophages (TAMs) play a role in PDAC as these suppress antitumor responses, promote metastasis and angiogenesis. TAMs are classified as either classical activated (M1) or nonclassical activated (M2). M1 cells have a proinflammatory and cytotoxic behavior, whereas M2 have the opposite functions and are thus pro-tumoral. M2 type TAMs and regulatory T-cells are accumulated in PDAC (Bulle et al., 2020). Pancreatic stellate cells (PSCs) are myofibroblasts and are responsible for fibrosis and desmoplastic reactions in PDAC. In a diseased pancreas, PSCs become activated, produce laminin, collagen, and fibronectin, and promote immunosuppression. In addition, an imbalance between PSCs that secrete matrix metalloproteinases (MMPs) for fibrosis repair, inhibitors of those metalloproteinases (TIMPs) and increased ECM production for tumor proliferation is observed. Fibroblasts present in the PDAC TME differentiate into cancer-associated fibroblasts (CAFs) upon TGF-β, EGF, FGF or TNFα secretion, hypoxia and oxidative stress. In turn CAFs secrete factors, which promote tumor growth and act as a barrier for drug delivery into the tumour site. There is a dynamic exchange of supportive signaling factors between CAFs and tumor cells. Two distinct subtypes of CAFs can be distinguished: myofibroblastic CAFs (myCAFs) and inflammatory CAFs (iCAFs). myCAFs express α-smooth muscle actin (α-SMA) and are located in the acinus, while iCAFs lack α-SMA expression, but express IL-6 and are more distantly located (Li et al., 2020). IL-6 can activate fibroblasts for ECM production. Blocking IL-6 in combination with chemotherapy, induced apoptosis of tumor cells and increased survival in mice (Long et al., 2017). Therefore, distinguishing between iCAFs and myCAFs is necessary before including these into drug screening tumor models due to different gene expression profiles and antigen presentation (Oehlund et al., 2017; Elyada et al., 2019). CAFs also support exosome release, which in turn increase chemoresistance-inducing factor when exposed to chemotherapeutics and secrete fibroblast activation protein α (FAPα) leading to angiogenesis and invasion (Kawase et al., 2015). Costa-Silva and colleagues demonstrated that exosomes derived from lesions are involved in liver niche formation. Uptake of the exosomes by liver Kupffer cells (KC) support the inflammatory state found in metastasis. High TGFβ level in patients is associated with poor prognosis (Costa-Silva et al., 2015), however when depleting CAFs, the TME was characterized by decreased angiogenesis, collagen deposition, increased cancer stem cell and regulatory T-cell numbers, and hypoxia, which resulted in worse patient survival (Özdemir et al., 2014). However, Ware et al. showed, that gemcitabine effect was impaired in stroma rich PDAC spheroids compared to spheroids without stroma (Ware et al., 2016). On the one hand studies suggest the need for targeting the tumor stroma in addition to cancer cells for a successful PDAC treatment, while on the other hand contradictory studies show that the complexity of the tumor stroma and that experimental design must be carefully taken into consideration.

Altogether, recapitulating the complexity of this disease in one single *in vitro* model is challenging, and it is more likely that multiple versions of PDAC models that serially include different components will together aid to reveal and translate processes influencing PDAC growth and progression. Therefore, platforms that enable the gradual incorporation of different bio- and physicochemical features are relevant for the development of translational PDAC models.

## Current *in Vitro* Models Used for Drug Screenings

The 5 years survival rate of PDAC is less than 7% and there are limited *in vitro* models to study pancreatic cancer. PDAC radiation treatment trials have been halted as no clinical benefit was observed. Targeted therapy of patient specific mutations of KRAS, TP53, SMAD4, MLL3, TGFBR2 and CDKN2A has not worked but needs further investigation. Apart from the mentioned therapies, NTRK fusion inhibitors seem to provide an alternative as well. Although progress is being made, there is no treatment for patients that relevantly improves outcomes (Roth et al., 2020). Chemotherapy remains the gold standard in treatment, when surgical resection is not possible. The standard of care chemotherapeutics are Gemcitabine, Gemcitabine/nab-Paclitaxel, Oxaliplatin and FOLFIRINOX (5-Fluorouracil, Leucovorin, Irinotecan, Oxaliplatin). However, treatment with these chemotherapeutics prolongs the life of patients only with a few months and the average survival remains less than 1 year (Frappart et al., 2020).

Immunotherapy has increasingly become a treatment option for various cancer types. Despite several trials, PDAC remains unresponsive to immunotherapies. Strategies to combine immunotherapeutics with chemotherapeutics are currently being evaluated in clinical trials (Nywening et al., 2016; Pfirschke et al., 2016). Furthermore, numerous approaches such as cytokine therapy (IL-2, IFN, IL-15), therapeutic vaccines, agonistic and antagonistic antibodies, small molecule agonists, adoptive cell therapy and chimeric antigen receptor were tested in PDAC (Brahmer et al., 2012; Zambirinis et al., 2015; Yuki et al., 2020). However, all of these treatments have either failed in clinical trials due to adverse effects, or due to ineffectiveness of the drug in the complex human PDAC tumor microenvironment. Therefore, it is necessary to screen for new drugs for finding new hits, which not only prolong the life of patients, but also cure this disease. Several approaches have been made in PDAC cancer research: Hou et al. studied the effect of FDA/EMA-approved drugs on pancreatic cancer primary cells and identified 14 drugs, which had an effect in four types of cells in 3D culture models (e.g., Bortezomib, Carfilzomib, Romidepsin, Homoharringtonine, and Trametinib). Two CAF lines and two PDAC cell lines were grown as monocultures in 2D, but also grown separately as spheres under the absence of exogenous ECM components in 3D to determine treatment differences in 2D and 3D. It was shown that 3D culture models were more resistant to chemotherapy than 2D cultures, indicating the importance of selecting the correct model for drug screenings (Hou et al., 2018). Phan et al., 2020 used the mini-ring method upon plating single cells mixed with Matrigel to generate organoids in a ring shape around a rim in a 96-well plate and to test 240 protein kinase inhibitors. This mini-ring approach allows drug testing on a very low number of cells, and they could discern different treatment behavior between patients. (Phan et al., 2020). Moreover, Driehuis et al. studied the effect of 76 therapeutics on PDAC organoids, and showed that some drugs are only effective in a subset of patients with the same or similar mutation pattern. These authors proposed the necessity of a personalized approach for achieving effective tumor killing (Driehuis et al., 2019). These studies suggest the necessity of stratified drug treatment and therefore promote the use of organoids, which can be harvested from each patient and subjected to drug screening prior to patient treatment for finding the most effective therapeutic. Frappart et al. (2020) used patient derived organoid (PDO) and Patient derived xenograft (PDX) models to screen several FDA-approved drugs and proved in *in vivo* studies, that PDOs derived from PDX serve as an alternative to PDX (Frappart et al, 2020). All these experiments with improved model systems raise hope for patients to screen for new drug candidates, as all previously in clinical trials tested drugs have failed so far. However, a high-throughput screening of drugs has not been feasible yet, due to the limited amount of patient material and organoids. Thus, a preliminary sequencing of the DNA of patients and mutation status could give an indication of the type of drugs that should be tested on organoids and afterwards prescribed to a given patient. Moreover, Haque et al. recently discussed the impact of the heterogenous TME in PDAC and concluded that extensive model development is still needed for establishing efficient drug pipelines (Haque et al., 2021). Therefore, current *in vitro* model systems are lacking in translational value, and novel PDAC models that allows for rapid initial drug testing that provide a truly translational outcome for identifying novel patient treatment options are called for.

## Modelling PDAC: *In Vitro* and *in Vivo*

**FIGURE 2:**
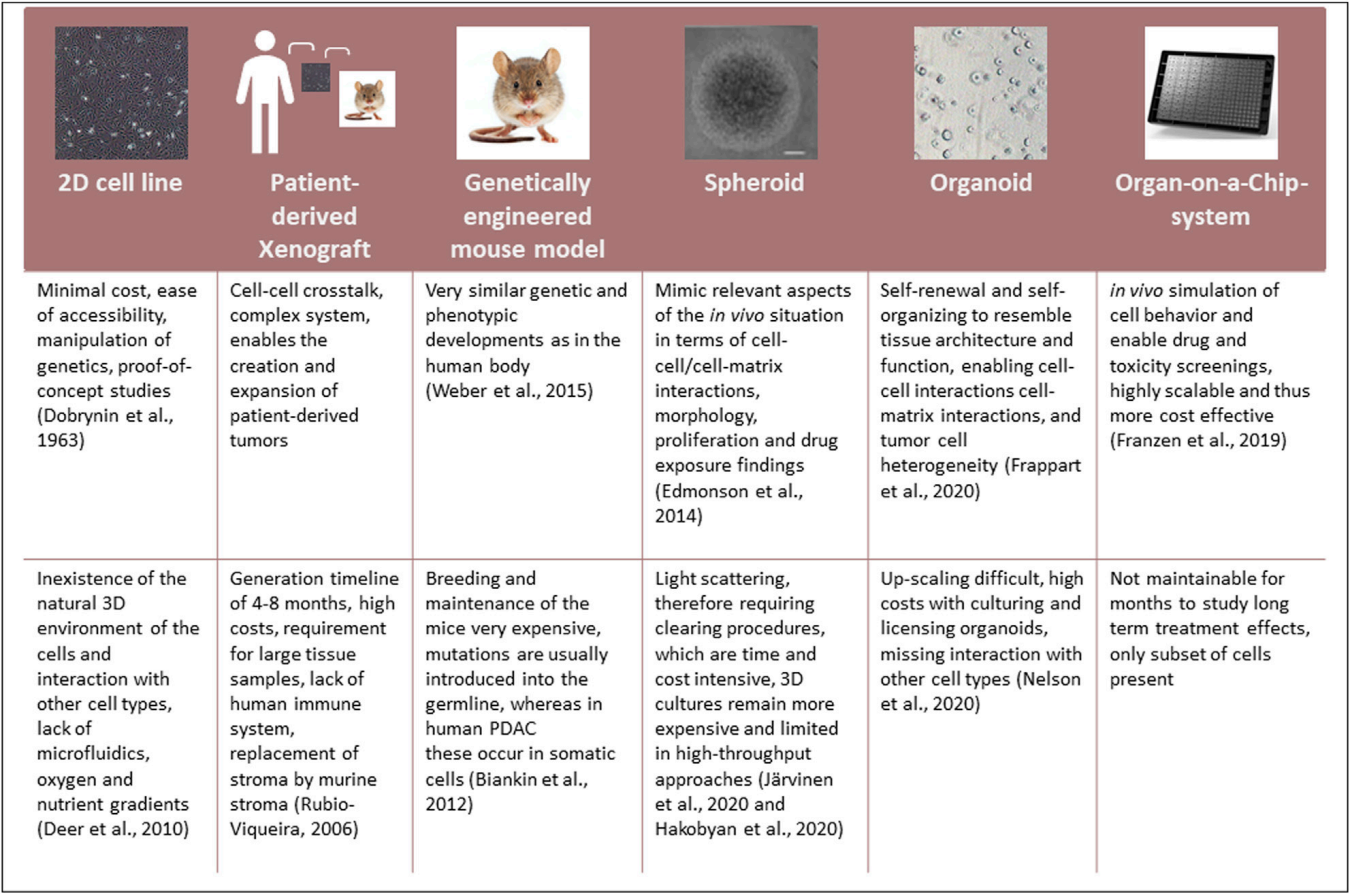
Comparison of the currently available model systems used in PDAC research.

Current drug screenings are mainly performed using conventional 2D grown PDAC cell lines, which were first generated in 1963 and remain used in research to date (Swayden et al., 2020). Nevertheless, several limitations influence the relevance of these models. Alternative model systems to cell lines grown in 2D have been established (Figure 2):

Every model has advantages and disadvantages. However, the use of organoids, especially in the context of Organ-on-a-Chip systems, is currently being widely regarded as potentially the models that most closely mimic the *in vivo* setting.

Organoids are three-dimensional structures recapitulating tissue geometry, dynamics, molecular and genomic signatures, thereby enabling *in vivo* like preclinical studies. Tumor organoids maintain patient-specific oxygen consumption, differentiation status and epigenetic marks as different patient samples showed a varied mutation status depending on diet, lifestyle and genetics and can thus contribute to tumor-related interpatient heterogeneity (Juiz et al., 2019). Organoids share stem cell characteristics such as self-renewal and can be maintained for several passages. Furthermore, organoids can self-organize to resemble tissue architecture and function, enabling cell-cell interactions, cell-matrix interactions, and tumor cell heterogeneity. PDX models and derived organoids have shown the same results in proteome analysis, however organoid models have proven less labor-intensive and more time efficient (Frappart et al., 2020). Another approach to use organoids would be to use genome engineering tools such as transcription activator like effectors (TALEN) or clustered regularly interspaced short palindromic repeats (CRISPR) in combination with a CRISPR-associated (Cas) 9 protein to introduce or excise mutations/genes, and to compare the phenotype and genotype of the newly generated organoid with a healthy wildtype organoid to study diseases (Schwank et al., 2013).

Although organoids can provide valuable translational models and potentially enable the development of personalized models, there are also challenges regarding up-scaling, and cost of culturing and licensing (Nelson et al., 2020). In addition, organoid cultures lack interactions with other cell types such as immune cells, vasculature, and stromal cells as well as biomechanical cues. Therefore, translational relevance of organoids is potentially limited when used in monoculture. Another relevant limitation is the variability across different organoid lines, genotypes, batches of organoids and even within the organoids in one culture. Even with these limitations, organoids have the potential to become the most valuable cellular model, that can be applied for patient stratification and personalized medicine in PDAC. To overcome lacking TME components, Frappart et al., 2020 introduced the term “PDACoids”, which entails the co-culture of PDAC organoids with immune cells and CAFs and further prompts the use of Organ-on-a-Chip approaches to incorporate several cell types (Frappart et al., 2020).

## Organ-on-a-Chip Systems

The majority of 3D cell culture systems do not incorporate multiple cell types such as endothelial, and stromal cells in addition to the PDAC cells and thereby fail to recapitulate the cellular complexity of the PDAC TME (Rothbauer et al., 2019). Thus, Organ-on-a-Chip systems provide novel platform for incorporating diverse cell types and flow conditions present in the human body. Moreover, tissue geometry, dynamics and gradients are recapitulated in specialized cell networks in these platforms (Bhatia et al., 2014). Organs-on-a-Chip are microfluidic systems, which enable the generation of a defined microenvironment for growing cells. As only a small number of cells is needed for experimental studies in Organs-on-a-Chip in comparison to traditional well plate cultures, it would be feasible to use these for patient stratification and for individualization of therapies (Van den Berg, 2019). The ductal tumor-microenvironment-on-chip (dt-MOC) applied by Bradney et al., gave further insight into the relevance of Organ-on-a-Chip systems in order to understand the complexity of intratumoral heterogeneity. EMT was mimicked by creating an epithelial cancer cell duct of KPC2, eKIC and mKIC mouse cell lines within a Collagen I matrix to study heterogenous invasion characteristics. The group thus generated a platform to study tumor cell invasiveness and aggressiveness and further suggested the development of patient-derived tumor-stroma interaction models (Bradney et al., 2020). As PSCs play a role in cancer progression, Lee et al. cultured tumor spheroids with PSCs in microfluidic devices to show, that the number of spheroids increased in co-culture conditions and that EMT related gene expression was increased as well (Lee et al., 2018). Another example of stromal cell incorporation is provided by Bi et al., who showed the successful incorporation of macrophages into a tumor-on-a-chip system, where they combined PDX generated PDAC cell lines with vasculature and immune cells. They demonstrate inhibition of tumor growth, invasion and angiogenesis in response to macrophages in their Organ-on-a-Chip device, which is a crucial step towards recapitulating TME complexity (Bi et al., 2020).

**FIGURE 3:**
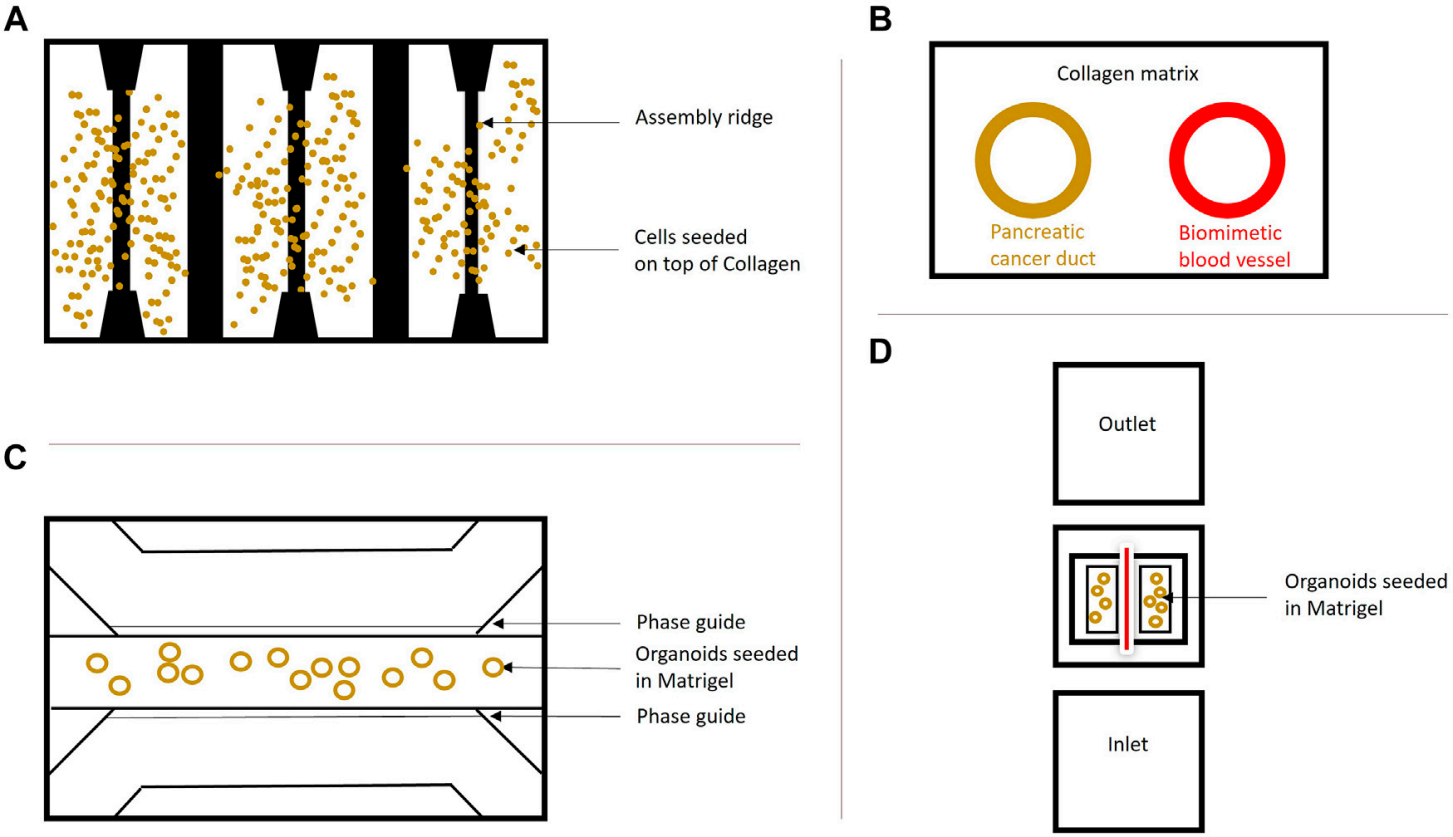
Organ-on-a-Chip systems. **(A)** Schematic overview of the HepaChip used by Beer et al. (2017). The chip contains eight culture chambers, fluidic inlet and outlet and gold electrodes to simulate hydrodynamics. The surfaces were coated with collagen and three different cell types were seeded and compared to 2D and regular 3D cultures of these cells. This model shows that organs grown on a chip resemble the *in vivo* drug treatment behavior better than cells grown in 2D and 3D and supports the findings by Kramer et al. (2019). **(B)** Schematic cross-section of PDAC-on-a-chip containing pancreatic cancer cells forming a duct and endothelial cells forming a biomimetic blood vessel by Nguyen et al. (2019). This system was employed to study vasculature in PDAC. A similar system (dt-MOC) was developed by Bradney et al. (2020), where an epithelial cancer cell duct was created within a Collagen type I matrix to study intratumoral heterogeneity. **(C)** The microfluidic 3-lane OrganoPlate^®^, based on a 384-well plate containing 40 individual chips with three channels for perfusion and ECM separated by PhaseGuides^TM^ can be used for growing Organoids on-a-Chip. The cells can be introduced into any of the three lanes depending on the purpose. Perfusion is ensured by the OrganoFlow^®^, which creates a height difference every few minutes at an adjusted angle to allow cell perfusion based on gravity leveling. This model was used by Kramer et al. (2019) to show a different treatment effect of chemotherapeutics in PDAC cells grown on a chip compared to monolayer culture. **(D)** Schematic InVADE platform used by Fook Lun Lai et al. (2020). Endothelial cells were suspended from the inlet and the outlet of the platform and unattached cells were removed upon perfusion. Attached HUVEC cells are represented in red, as they form a tube. The PDAC organoids were seeded in Matrigel within the middle chamber surrounding the endothelial cells. This model was established to show the importance of the tumor microenvironment in pancreatic cancer.

In addition, Organ-on-a-Chip models containing PDAC organoids can also be applied for studying drug efficacy in drug discovery and development programs. Mimetas developed several OrganoPlates (Figure 3) to establish 3D *in vitro* model systems on a chip, which do not require the introduction of perfusion loops between diverse organ compartments and ensure perfusion, vascularization, and high-throughput in a standard 384-well plate format (Beaurivage et al., 2019). Kramer and colleagues used S2-028 PDAC cells grown on a chip for gemcitabine treatment, which inhibits DNA synthesis and induces apoptosis. Therefore, the cells were cultivated in the 3-lane OrganoPlate^®^ and the group observed a different effect of gemcitabine treatment in monolayer culture compared to Organs-on-a-Chip, thereby suggesting an overestimation of the actual drug efficiency in 2D (Kramer et al., 2019) (Figure 3C). Moreover, the HepaChip^®^ with continuous perfusion of BxPC3, MiaPaCa2 and PANC1 lines was used by Beer et al. to show, that Organ-on-a-Chip platforms can be used for improved prognosis and drug testing, as their drug screenings initially proved, that *in vivo* drug responses are more closely mimicked with these platforms compared to normal 2D or 3D systems (Beer et al., 2017) (Figure 3A).

Also, vascularization can be studied in these platforms, as vascularization of the organoids is necessary to simulate *in vivo* conditions for waste removal and distribution of oxygen and nutrients (Huang et al., 2020). Another system called Integrated Vasculature for Assessing Dynamic Events (INVADE), based on a 96-well plate with inlet and outlet wells connected to a tissue chamber was used by Fook Lun Lai et al., 2020 in which human umbilical vein endothelial cells (HUVEC), human dermal fibroblasts and PDAC organoids were cultivated together. Subsequently, cytokine release, fluorescent dye distribution and viability after drug treatment for a better understanding of the TME in PDAC were studied. Fook Lun Lai et al., also observed a difference in organoid diameter and ECM remodeling between organoid monoculture and co-culture with fibroblasts. In addition, they also tested molecule transfer in monoculture and co-culture and concluded that the transfer from endothelial cell vessels is inhibited in the co-culture conditions, which might explain the role of the tumor stroma in insufficient chemotherapeutic treatment of PDAC (Fook Lun Lai et al., 2020) (Figure 3D). Ngyugen et al. used a similar setup and included a HUVEC vessel and primary mouse pancreatic cells in their model, to show a highly complex cellular interaction network, invasion of PDAC cells into the vasculature, thereby contributing to a better understanding of metastasis in PDAC with an Organ-on-a-Chip platform (Nguyen et al., 2019) (Figure 3B).

In addition, higher throughput can be achieved with these platforms as described by Drifka et al., as their tubing-free and easy-to-load microfluidic device opens the possibility for implementing automated liquid handling. Moreover, they also discuss the importance of the PDAC TME for incorporation of the *in vivo* complexities of 3D architecture and cell-cell interactions within a model containing primary PSCs and the PANC-1 human PDAC line (Drifka et al., 2013). Most Organ-on-a-Chip platforms reduce media and reagent consumption, are compatible with most laboratory equipment and the systems are often tube- and pump-free, allowing for the adoption of these platforms in many research labs. Thus Organ-on-a-chip platforms are revolutionizing research upon enabling tumor microenvironment studies in a high-throughput setting. Other advantages of Organ-on-a-Chip systems include a better mimicking of cell behavior and easier drug efficacy and toxicity screenings, which can in the long term enable treatment stratification and individualization. Moreover, this would also decrease the costs and duration of drug development. As research and development costs will decline upon fewer experiments needed in preclinical development due to better predictability, economic impacts will be lower during drug development studies with approximately 10–26% cost reduction. This would save several hundred million dollars per drug on the market (Franzen et al., 2019).

Although Organ-on-a-Chip platforms potentially provide an excellent tool to simulate the *in vivo* situation as closely as possible, there is still need for further development. A whole-body set-up as in animal models has not been modelled so far and thus physiological effects cannot be studied with current Organ-on-a-Chip platforms. Another limitation is that cultures cannot be maintained for a long time period which would be necessary to study long term treatment effects. In addition, there is still only a subset of cell types present, which does not fully recapitulate the cell-cell interaction and signaling within certain tissues. Moreover, high throughput of existing platform is not comparable to well-plates and thus this system will still find its main application in low throughput personalized patient treatment. Low throughput personalized treatment comprises patient cell sample isolation, expansion, Organ-on-a-Chip loading of cells and drug testing based on a patient’s mutation status to quickly identify drug candidates (Esch et al., 2015). Thus, the selected treatment would be matched to a patient’s disease status and thus treatment success can be guaranteed earlier as no other treatments have to be tested first in patients, thus potentially impacting patient survival. However, given the potential application of translational Organ-on-a-Chip based models, these platforms will likely in the long-term positively impact drug discovery and development programs making these more efficient and likely more cost effective.

As the technology matures and more scientists begin to use Organ-on-a-Chip platforms more data relevant for preclinical studies will become available and help improve the current platforms (Van den Berg et al., 2019).

## Discussion

PDAC remains one of the deadliest cancer types due to limited diagnosis at an early stage, multidrug resistance and its high metastatic potential into the liver and lung. Therefore, there is an urgent need for *in vitro* models that recapitulate tumor behavior more accurately and potentially also model patient specific responses. Organoids provide an outstanding tool for drug screening approaches in both academic proof-of-concept studies as well as in preclinical studies for replacement of 2D cell culture systems and animal testing. As numerous studies have suggested, morphological as well as functional (gene expression) differences occur between 2D and 3D models, better alternatives to 2D models are needed and organoids might in part overcome the gap between *in vitro* and *in vivo* responses. Also, for drug discovery programs as well as for personalized medicine it is necessary to be able to not only study intratumoral but also intertumoral heterogeneity, that exists between the diverse cell types and different patients.

The establishment of a “PDACoids” 3D system, which incorporates many different cell types and uses a synthetic matrix such as a hydrogel, thus eliminating batch-to-batch variation and potential pathogen transfer, is an important step to make personalized and preclinical research using organoids more robust (Dutta et al., 2017). Organoid experiments can be further expanded in Organ-on-a-Chip systems, which allow the incorporation of fluid flow and stromal cells for better tumor disease modelling. The major limitations of Organ-on-a-Chip platforms in personalized medicine approaches result from limited access to patient samples and corresponding clinical data as well as from relatively low-throughput and lacking automation of most Organ-on-a-Chip platforms.

Thus, PDAC-on-Chip models will likely evolve into low/medium-throughput platforms that will enable drug response studies and contribute to further development of the PDAC therapeutic field. Relevant PDAC models should include PDAC organoids that represent different patients in combination with a stromal cell compartment, represented by pancreatic stellate cells, cancer associated fibroblasts, and immune cells. The incorporation of lymphocytes into PDAC models is important to reveal potential reasons for failures of immunotherapeutic strategies and to model T cell function. The introduction of NK cells, DCs and macrophages might further allow to study receptor interactions and to provide a more complex *in vivo* like system. Also, the inclusion of endothelial cells for angiogenesis and lung and liver organoids for studying metastasis would favor a good simulation of the PDAC TME in conjunction with processes that impact tumor progression. Organ-on-a-Chip platforms could revolutionize PDAC research if improvements are made. High-throughput is still a limitation for most Organ-on-a-Chip platforms, whereas scalability of these assay units and automation are crucial for enabling larger drug screenings. Moreover, pump and tubing free methods whilst still assuring fluid flow will pioneer over their competitors, as these require lower maintenance, less time and are easier to handle. These platforms should also incorporate as many cell types as feasible to create a translational model and potentially replace animal models in the near future.
